# Supplementary material for: Are carbon emissions trading and green financial instruments synergistic? -Comprehensive quantitative research based on content analysis
Source: PLoS One. 2024 Mar 7;19(3):e0298601. doi: 10.1371/journal.pone.0298601 (PMC10919601; doi:10.1371/journal.pone.0298601)
Supplement: S1 Data — (ZIP) [file pone.0298601.s001.zip › Data/地级市Z_F工作报告.zip!/░▓╗╒/┴∙░▓╩╨/└┤╫╘╣1⁄2╓┌║┼í╛┬φ┐╦╩2╛▌═°í┐_2004.docx]

各位代表:

　　现在，我代表市人民政府，向大会作政府工作报告，请予审议。同时，请市政协委员和其他列席人员提出意见。

**一、2003年工作回顾**

　　2003年，在市委的坚强领导下，全市人民同心同德，顽强拼搏，战胜了非典和特大洪涝灾害造成的困难，实现了经济稳步增长和社会事业明显进步。

**国民经济继续保持增长。**全年实现国内生产总值208.1亿元，比上年增长5.6%。其中，一产增加值65.4亿元，下降5.7%；二产增加值72.1亿元，增长14.1%；三产增加值70.6亿元，增长9.6%。财政收入14.5亿元，增长3.4%，其中地方财政收入10.4亿元，增长3.3%。城镇居民人均可支配收入5139元，增长7.2%。农民人均纯收入1736元，下降1.6%。全社会固定资产投资额66.7亿元，增长16.8%。社会消费品零售总额89.1亿元，增长7.5%。外贸出口额1.03亿美元，增长25%。民营经济实现工商税收3.6亿元，增长39.3%。

**抗击非典和抗洪救灾取得阶段性胜利。**针对非典疫情，及时建立预防和应急救治体系，构筑覆盖城乡的群防群控网络，保证了全市无确诊病例和疑似病例。面对特大洪涝灾害，全力组织抗洪抢险，安全转移43万群众，确保了淮河大堤、五大水库、主要内河堤防和城市防洪安全。汛情稍有缓解，及时转移工作重心，大力开展生产救灾和灾后重建，妥善安排灾区群众基本生活。截止去年底，累计发放救灾款8651万元、蓄洪区运用补偿资金1.7亿元，开仓借粮2054万公斤，募集衣被128万件（床），完成灾民建房65347户、137285间，移民迁建4183户、7729间，修复重建水毁校舍45.3万平方米，先后派出医疗小分队2000多支，免费诊治灾民30万人次，保证了灾区群众有饭吃、有衣穿、有房住、无疫情。

**重点经济工作成效明显。**招商引资：全年到位境内资金25.4亿元，增长113 %；利用境外资金3136万美元，增长34.8%；长江精工工业园、华电六安电厂等一批重大项目落户我市。企业改革发展：列入省考核的116户国有集体中小企业，有98户完成改制任务；市属重点国有工业企业基本实现改制退位，置换国有资产3.48亿元、职工身份5506人；全市规模工业实现增加值27.6亿元、利税6.3亿元，分别增长12.3%和15.7%，创建市以来最好水平；开发区和工业园区建设力度加大，全年投入六安开发区基础设施建设资金1.2亿元，相当于建区10年的总和。农业产业化和结构调整：组织实施了农业产业化“128工程”，庆发集团进入国家级、源牌集团等11家企业进入省级农业产业化重点龙头企业行列；“六安瓜片”、“霍山黄芽”获国家证明商标；11个农产品被认定为绿色食品，41种农产品被认定为无公害产品；非种植业比重上升到64.7%；主要农产品优质化率达55%；劳务输出138万人，收入增长26.9%，对农民现金收入增长的贡献率达70.6%。城市建设：城市总体规划修编完成；滨河公园一、二期工程，火车站广场及站前大道和高架桥，淠河防洪堤和古城墙，梅山中路、万佛路一期和解放北路配套等工程相继建成，污水处理厂通水试运行，皋城东路主车道建设和梅山北路延伸、磨子潭路改造工程基本结束，光明东路、龙河西路、经三北路建设和解放中路单边拓宽配套工程加紧施工，中心广场和行政中心建设全面铺开；城市公共设施建设不断加强，绿化、亮化工程取得初步成效。

**基础设施建设步伐加快。**宁西铁路六安段投入运营; 105国道马（店）姚（李）段改造通过验收，商景高速公路六安段开工建设，宁西高速公路大（顾店）叶（集）段封闭改造提前施工。投资3.5亿元改造县乡公路，完成105个村道路配套建设。临淮岗工程提前一年成功截流，城西湖大堤和佛子岭、磨子潭水库除险加固进展顺利，淮河及淠河、史河等内河治理项目争取和建设工作成效明显，江淮分水岭综合治理开发和人畜饮用水工程取得新的进展。

**就业再就业和社会保障工作得到加强。**全年新增就业岗位2.7万个，其中1.5万名下岗失业人员和1442名“4050”人员实现再就业，城镇登记失业率4.1%。国有企业下岗职工基本生活费和企业离退休人员养老金基本做到按时足额发放；为2万多名登记失业人员发放失业保险金3010万元。全市基本养老保险、失业保险、医疗保险参保人数分别达16.9万人、22.2万人和18.3万人，征缴社会保障基金突破3亿元。全年纳入城镇最低生活保障对象7.6万人，发放低保金3427万元，基本做到应保尽保。扶贫开发和优抚对象及残疾人、五保户、特困户等弱势群体的保障、救助工作进一步加强。

**精神文明建设和社会各项事业有新的进步。**群众性精神文明创建活动深入推进。科技推广、普及工作广泛开展。基础教育和职业教育稳步发展，完成中小学校危房改造38.2万平方米。人口与计划生育、环境保护和土地管理三项基本国策进一步落实，人口出生率控制在12‰以内。文化、卫生、体育、新闻出版、广播电视等社会事业取得新的成绩。妇女儿童和老年人合法权益得到进一步保障。民族、宗教、外事、侨务、对台、审计等工作得到加强。市政府成立了决策咨询委员会，启动了新闻发布会制度，开展了政风行风评议和人民建议征集活动。国防后备力量建设和民兵预备役工作进一步加强，全民国防教育和“双拥”工作深入开展。“四五”普法教育扎实推进。社会治安综合治理工作进一步加强，社会秩序保持稳定。

　　各位代表，2003年，我们在十分困难的情况下取得这样的成绩，的确来之不易。这得益于市委的正确领导，得益于人大、政协及社会各界的大力支持，得益于全市人民的团结奋斗，得益于原地委、行署和市委、市政府多年来奠定的工作基础。在此，我谨代表市人民政府，向全市广大干部群众，向各位人大代表、政协委员和各民主党派、人民团体，向驻六安的人民解放军、武警官兵和中央、省属各单位，向所有关心、参与、支持六安改革开放和现代化建设事业的同志们、朋友们，表示衷心的感谢和崇高的敬意！

　　在肯定成绩的同时，我们也清醒地看到，经济社会发展中还存在不少问题，主要是：结构性矛盾突出，非公有制经济和第二产业比重以及城镇化率偏低；招商引资总量较小，引进大项目和利用境外资金偏少；工业支柱产业的支撑力、牵动性不强，少数骨干企业效益下滑；农民增收困难，灾区群众生活水平下降，因灾、因病返贫现象比较严重；城市管理与建设不够协调，基础设施建设相对滞后；城市就业再就业面临较大压力，社会保障资金缺口较大；财政收入增长缓慢，收支矛盾突出，多数乡镇村债务沉重，运转困难；市场经济秩序仍不规范，损害群众利益的现象时有发生，社会不稳定因素依然较多；特别是国内生产总值、财政收入和农民人均纯收入未达到预期目标，国民经济中几个主要指标增幅均处于全省后位。存在这些问题，既有灾害影响和自身条件的制约，也反映出我们的工作还存在许多差距。主要是思想解放不够，改革创新的步子不快，抓落实的力度不够大，政府的职能转变不到位、服务不优等。在新的一年里，我们一定要采取有力措施，认真加以解决，千方百计加快改革和发展的步伐。这是全市人民的迫切愿望，也是各级政府义不容辞的职责。

**二、2004年工作的指导思想和奋斗目标**

　　2004年是实施全面建设小康六安“三步走”战略的第一年，也是完成十五计划的关键一年。我们既面临世界经济复苏、国内经济进入新一轮周期上升阶段的难得机遇，又面临国际、国内区域和产业竞争日趋激烈的严峻挑战；既有近年来自身积累的良好发展基础，又有去年自然灾害造成的严重滞后影响。我们必须进一步认清形势，把握机遇，趋利避害，扎扎实实做好各方面的工作，促进经济社会更快、更好地发展。

　　今年政府工作的指导思想是：**以邓小平理论和“三个代表”重要思想为指导，按照市委一届六次全会关于全面建设小康六安“三步走”战略的总体部署，坚持以民为本的执政理念不动摇，坚持工业化核心战略和招商引资主战略不动摇，坚持把“三农”工作放在重中之重的位置不动摇，努力实现招商引资、工业改革发展、农业产业化和结构调整、城市重点工程建设的新突破，进一步加强民主法制建设和精神文明建设，促进经济持续快速健康协调发展和社会全面进步。**

　　为确保全面建设小康六安“三步走”战略目标的顺利实现，从今年起，全面启动“568”计划，即打造以绿色食品为主的优质农产品基地，以食品及农副产品深加工、机械、建材、医药化工、轻纺为主的加工制造业基地，以火电和水电为主的能源基地，以钢铁生产为主的冶金基地和以“红色、绿色”和其它特色为主题的旅游基地等5大基地；在未来五年内，以招商领头、五路跟进（启动民间资金，调动金融资金，争取政策性资金，抓好经营城市，推进资本运营）筹集资金，实施华电六安电厂建设、铁矿开发及钢厂建设、高速公路建设、“三农”工程建设、中心城市及县城建设、开发区及工业园区建设等6个超100亿元的重大综合建设项目；确保2010年实现人均国内生产总值800美元以上的目标。

　　今年经济和社会发展的主要预期目标是：国内生产总值增长10%左右；财政收入增长10%左右；城镇居民人均可支配收入增长8%左右；农民人均纯收入增长8%左右；新增城镇就业岗位2.35万个，城镇登记失业率控制在4.5%以内；全社会固定资产投资额增长20%左右；社会消费品零售总额增长8%左右；进出口总额增长10%左右；境外引资增长30%左右，境内引资增长20%左右；人口自然增长率控制在6.9‰以下。

　　围绕上述指导思想和奋斗目标，今年将着力实施“六大工程”，即以实现速度质量效益相统一、经济跨越式发展为目标的经济振兴工程；以保证城乡困难群众生活无饥寒、子女能上学、生病可就医、权益有保障为目标的扶贫济困工程；以提高人民群众思想道德和科学文化素质为目标的提升素质工程；以创造优良生活、工作和投资环境为目标的环境优化工程；以确保治安秩序良好、社会稳定为目标的六地平安工程；以提高政府决策和施政水平为目标的阳光政务工程。

**三、加速推进工业改革发展**

　　实施工业化核心战略，走新型工业化道路，深入开展“发展工业学霍山”活动，确保规模工业增加值增长12%以上，利税同步增长，加速确立工业在全市国民经济中的主体地位。

**做大做强支柱产业和骨干企业。**以大企业、大集团、大项目为核心，立足资源优势、比较优势和现有产业，发展壮大冶金、汽车零部件、机电化工、食品等雏形较好、牵动性强的链型产业，整合提升羽绒、柳编、铸造、小水电、木竹加工等有一定规模和优势的块状产业，加快形成区域核心产业群。择优扶持30户左右骨干企业，实施资产重组，推进挂靠联合，促其做大做强。力争培育年销售收入超5亿元企业1-2家，超3亿元企业5-6家，超2亿元企业7-8家，超亿元企业15家。

**大力发展中小企业。**围绕优势资源和农副产品的加工、转化与增值，培植一批有发展潜力的资源加工型、劳动密集型和出口创汇型中小企业。坚持错位发展战略，主动承接外地大企业的产品扩散，发展配套和协作经济，培育“专、精、特、新”中小企业集群，力争新增规模工业企业60户以上。

**深化完善企业改革。**进一步深化国有工业企业改革，对尚未进行产权制度改革的企业进行彻底改制。开展企业改革“回头看”活动，按照国有资本退出、职工身份置换、存量资产盘活和金融债务处理“四个到位”的要求，对已改制企业进行规范完善。推动骨干企业深化内部改革，完善法人治理结构，加快建立现代企业制度。帮助世林照明公司扎实做好上市辅导工作，争取早日上市融资。

**加快技术进步和管理创新。**坚持用先进适用技术改造、提升传统产业，实施应流集团精炼炉生产线等5个投资5000万元以上重大项目，双龙公司数控机床等10个投资3000万元以上重点项目，星星轻纺棉制品生产线等25个投资1000万元以上骨干项目，确保技术改造投资增长15%以上。加强长江股份、龙津集团等5家省级企业技术中心建设，支持企业开展共性、关键性技术攻关，组织实施将军磁业微波磁体等6个技术创新项目。全年力争开发新产品200个，其中省级新产品20个以上。加快推进企业战略管理和信息化建设，实施ISO等标准体系认证，改善和加强企业基础管理。

**四、全力实现招商引资新突破**

　　围绕招商引资主战略，突出工业招商，坚持内外资并举，大中小并举，多产业并举，主攻长三角、珠三角地区，拓展港澳台、日韩等国家和地区，努力实现大企业招商、大项目招商和直接利用外资新突破。全年实际利用境内资金30亿元以上，境外资金4000万美元以上，工业和农业产业化方面的引资额力争达到引资总额的60%以上。

**构筑招商引资平台。**加快开发区建设与发展。六安开发区要按照“生态园、科技园、工业城”的定位，培育主导产业，发展关联产业。实施 “五路三桥”工程，力争实现3平方公里的“七通一平”、国内生产总值3亿元、财政收入3000万元目标，引进10个1000万元以上项目。叶集开发区要力争基础设施投入和引进资金“双过亿”。加快工业园区建设，积极吸引外资、启动民资参与园区开发。按照“开放式运营、封闭式管理、一站式服务”的要求，创新园区管理和运作方式。力争全市工业园区实现基础设施投入10亿元、项目到位资金10亿元目标。

**强化招商引资项目工作。**围绕特色资源、主导产业和存量资产，编制一批牵动性强、关联度高的重大项目，特别是加工制造业项目。市项目库储备投资规模3000万元以上项目不少于100个，县区项目库储备1000万元以上项目不少于50个，重点项目和即期启动的项目要达到可研报告或商务计划书深度。对现有项目按产业分类，整合包装，形成板块，集中推介。同时，积极做好华电六安电厂二期项目争取工作，加快实施安徽北大科技园、特种钢铁企业等重大引资项目。

**创新招商引资机制。**坚持全民招商，强化企业招商和专业招商，推行挂职或离岗招商，发展代理招商，注重以商招商，组织和参加节会招商。进一步强化招商引资目标管理，完善考核体系，严格兑现奖惩。健全项目跟踪落实机制，继续实行重大项目领导联系制度。强化督促检查机制，实行招商引资联席会议、按月通报调度和定期巡回观摩检查等制度。

**改善投资环境。**进一步完善市行政服务中心、招投标中心、招商洽谈中心、投诉受理中心和国库集中支付中心建设，年内实现集中办公。建立健全“一站式”服务工作机制。严格实行执法人员“六不准”制度。设立经济环境63110投诉专线，强化投诉案件查处工作，切实维护外来投资者合法权益。聘请部分人大代表、政协委员和外来投资者担任监督员，对招商引资服务质量和执法情况进行定期测评和年度评议，结果在新闻媒体上公布。

**五、积极促进农民增收和农村经济全面发展**

　　以增加农民收入为核心，坚持 “多予、少取、放活”，用工业的理念抓农业，统筹城乡发展，加快农村奔小康进程。

**调整优化农业结构。**按照高产、优质、高效、安全的要求，以优质安全农产品基地建设为抓手，以畜牧水产业和高效经济作物、园艺作物为重点，加强示范引导和服务，促进农业产业结构调整。重点抓好9个无公害农产品示范基地建设。大力发展以皖西白鹅、奶牛、山羊等食草畜禽和名优水产品为重点的养殖业。优化农业区域布局，根据山库区、江淮丘陵和平原圩畈的现实条件，建设多种类型的农业园区。加大优良品种引进、推广力度，主要农产品优质化率达到60%以上。深化农业技术推广体制改革，健全市场信息服务体系、农产品质量标准和检验检测体系。加强动物防疫检疫，精心组织好高致病性禽流感防治工作。

**推进农业产业化经营。**围绕优质粮油、名优茶叶、畜禽水产、草竹柳编、安全果蔬、茧丝绸等优势产业，整合相关资源，择优扶持一批带动力强、辐射面广的龙头企业。大力发展农村中介组织，特别是农民专业合作组织。推进品牌整合，扩大现有15种安徽名牌农产品生产规模，培育一批富有六安特色的市级名牌农产品。加强农产品市场和营销网络建设，改造茶叶、粮油、木竹、板栗、羽绒等传统市场，建设绿色农产品市场；积极发展以超市为平台的农产品连锁专卖，进一步拓展“六安绿色之窗”销售网点，发挥农网作用，搞活农产品流通。

**引导农民双向创业。**引导农民在农村创业，开发现代农业；引导农民去城市创业，发展劳务经济。加快城乡统一的劳动力市场建设，提高劳务输出组织化程度。抓好劳动力的职业技能培训，加强外出务工人员的跟踪服务，在北京、上海、广州等我市民工集中地区设立工作站，帮助务工人员妥善解决工资兑现、子女就学、权益保障等方面的问题。组织开展“2004·六安民工维权救助行动”。重点建立1000名优秀创业者档案，鼓励事业有成者回乡创业。

**落实党在农村的各项政策。**深化农村税费改革，认真落实各项配套措施，加大涉农案件查处力度，严防农民负担反弹。严格按照国家规定的减税比例把农业税调减到户，真正使农民得到实惠。深化粮食流通体制改革，完善粮食补贴方式改革，保证粮补资金直接到户。稳定农村家庭承包责任制，引导农户土地承包经营权依法、自愿、有偿流转，逐步发展适度规模经营，培育庄园经济。实行最严格的耕地保护制度，严禁非法侵占农民承包地，依法保护失地农民的合法权益。加快小型水利设施产权制度改革，引导社会办水利。严格控制并积极稳妥地化解乡镇村债务。

**六、继续推进城镇化和基础设施建设**

　　坚持优先发展六安中心城市，带动县城和中心镇建设，强化产业和人口集聚功能，为经济和社会发展创造良好条件。

**加快中心城市建设。**按照改造老城、拓展新区、路网先行、拉开框架、调整布局、完善功能、突出特色、提高品位的要求，加快推进六安中心城市建设。继续坚持经营城市、滚动发展的理念，实行政府、企业、社会多渠道筹资；深化城市公用事业建设和管理体制改革，全面推行环卫保洁、道路冠名、户外广告等市场化运作。通过经营城市和运用金融资金，力争城市建设资金投入20亿元。加快行政中心、中心广场、人民广场建设，完成光明东路、淠河路防洪墙等在建工程，新建长安南路、佛子岭西路等10条道路；改造解放中路、皖西东路等8条道路；实施淠河、淠河总干渠城区段二期治理；加快西都时代广场等大型商场建设，规划建设商业步行街；搬迁建设汽车客运站场；完成常青路和皋城广场、云露桥、大雁河、古城遗址的部分整治；开工建设体育中心场馆、妇女儿童活动中心，规划建设皋陶陵园、大戏院、博物馆、老年活动中心。配套建设库容量380万立方米的城市生活垃圾填埋场、5个垃圾中转站和10座公厕等公用设施，启动污水管网三期工程，新增城区天然气用户5000户。规划建设20万平方米拆迁居民安置房，切实解决困难拆迁户的安居问题。

**提高城市管理水平。**建立“两级政府、三级管理、四级网络”管理体制，逐步形成全社会管理、全过程控制、全方位覆盖的长效管理机制，提高城市管理效能。做好社区发展规划，加强社区建设，创建一批标准化社区。严格城市规划管理，强化工程建设管理，组织开展城市建设工程用户满意度公开评议活动。加强地名设标工作，推进地名标准化建设。继续实施绿化、净化、亮化、美化工程，加强城区交通秩序管理，加大市容市貌综合整治力度，突出整治摊点群，切实解决“脏、乱、差”问题，为市民创造最佳人居环境。

**推动县城和中心镇建设。**各县区要确立经营城市的理念，加快县城基础设施及其配套设施建设，增强其载体功能。改革户籍管理制度，引导和鼓励农村人口向城镇聚集。叶集试验区要依托区位和体制优势，加快城市化进程。各中心镇要完善建设规划，走以产业兴城镇、以城镇促产业的路子，发展特色经济，带动规模扩张，逐步形成产业发展、人口聚集、市场扩大的良性互动机制，加速农村城镇化步伐。

**加大基础设施建设力度。**加快建设宁西高速公路六安段（93公里）、商景高速公路六安段（185公里），争取开工建设合武高速公路六安段（90公里）、合阜高速公路六安段（11.5公里），改建国省道路454公里，建设县乡道路687公里。基本建成临淮岗洪水控制工程，开工建设白莲崖水库，兴建一批淮干整治、行蓄洪区及内河治理、城市防洪和大中型水库除险加固项目。加强农田水利基本建设和小流域综合治理，深入推进江淮分水岭综合治理开发。加强农村能源建设。实施六安22万伏第二变电所和霍邱高塘、叶集两个11万伏输变电工程，改善供用电条件；实施通信“村村通”工程，努力改善边远地区和山区通信状况。

**七、扶持壮大民营经济**

　　进一步解放思想，放开搞活，促进民营经济扩大规模，提升素质，尽快成为市、县区经济的主体。

**拓宽民营经济发展领域。**坚持“非禁即入”原则，进一步完善发展民营经济的激励政策，引导民营资本投资基础产业、现代服务业和教育、文体等社会事业，参与建设交通、能源、水利等基础设施和城市公用事业；鼓励民营企业收购、兼并、控股、参股、租赁国有、集体企业。拓宽民营企业创业通道，改企业注册审批制为登记备案制，允许新办民营企业注册资金分期到位，实施预备期管理。民间资本投资，享受招商引资政策。鼓励机关、事业单位工作人员按规定领办民营企业，允许机关、事业单位工作人员依法投资入股民营企业，积极引导和鼓励转业退伍军人、下岗失业人员、大中专毕业生投身民营经济。

**扶持优势民营企业。**选择20户左右民营骨干企业，进行重点扶持，培育具有较强带动力的民营企业集团。鼓励民营企业通过招商引资、资本运营、品牌合作、协作配套等形式快速扩张。积极引导民营企业加快体制、技术和管理创新，提高科技和管理水平。大力支持具有比较优势的中小民营企业加快发展，形成各具特色的民营企业群体。

**完善民营经济服务体系。**拓宽民营企业融资渠道，增加对民营企业的信贷投放；健全中小企业信用担保体系，扩大担保规模。加强对民营企业科技创新能力的培养，促进民营科技企业发展。重视面向民营企业的技术、管理培训，提高其技术开发、市场营销和资本运营能力。坚决取消对民营企业的各种违规收费和违规检查，保障其合法权益。引导和促进民营企业诚实守信、合法经营。

**八、大力发展第三产业**

　　以市场化、产业化为目标，提升传统服务业，发展现代服务业，努力提高第三产业在国民经济中的比重。

**积极推进商贸流通业改革发展。**以民营化为目标，上半年基本完成国有商业企业改革任务。充分利用现代技术和管理手段，加快构筑物流配送、交通运输、公共信息三大平台。抓住宁西铁路开通和高速公路建设的机遇，规划建设一批大型购物中心和区域性消费品市场、生产资料市场；发展连锁货仓、代理、配送等流通业态，培育壮大一批连锁经营企业。积极发展第三方物流等新型流通方式。扩大交通运输设施规模，推动运输业加快发展。以发展电子政务和电子商务为突破口，提高信息网络技术的应用和普及水平，努力打造“数字六安”。

**进一步扩大对外经济贸易。**实施以质取胜和市场多元化战略，优化出口商品结构，拓展国际市场，扩大出口规模。主动适应国家政策调整，加强出口退税工作。对出口创汇超500万美元的企业，予以重点扶持。进一步扩大生产企业特别是民营企业进出口经营权。推动有条件的企业“走出去”，开展跨国经营。针对我国入世过渡期大部分保护措施到期的新情况，及早准备应对预案。

**大力发展旅游业。**以“红色、绿色的大别山”为主题，以革命传统教育和观光、休闲为重点，加强与周边地区合作，编制大别山旅游规划，加快旅游资源开发。注重研究开发以皋陶文化、楚汉文化、红军文化为代表的特色旅游项目。加强旅游基础设施和旅游业队伍建设，招商新建两座四星级以上标准宾馆。主动与大中城市旅游网络对接，加强旅游资源的整合、包装和推介，积极推进旅游景点经营权转让，吸引各种资本参与旅游资源和产品开发，做大旅游经济。

**规范发展房地产业和社区服务业。**坚持积极引导、适度调控、规范管理，促进房地产业健康发展。积极发展房地产二级市场，建立健全公开、公平、公正的房地产交易机制。规范发展物业管理和房屋租赁市场。加强社区公共设施建设和社区服务，大力发展家政、维修、托幼、养老、保健、餐饮、娱乐以及安全防范等社区服务业。同时，注重培育会计、律师、咨询、经纪等各类中介服务组织，发展土地、技术、劳动力等要素市场。

**积极做好财税金融工作。**坚持开源节流，加强税收征管，实现财政收入稳定增长；强化预算管理，厉行节约，按照保吃饭、保运转、保稳定的要求，科学安排支出，加大对“三农”、教育、科技和社会保障的投入。深化公共财政支出改革，进一步完善国库集中支付制度，扩大政府采购范围。全面推行“乡财县管”改革。继续开展创建金融安全区活动。加强银政、银企联系与合作，引导金融机构加大对地方经济的信贷支持。加快组建城市商业银行，构筑地方金融支撑体系。深化农村信用社改革，鼓励有条件的县区组建农村合作（商业）银行。大力推进小额信贷，加大对“三农”的信贷支持。

**九、切实解决关系人民群众切身利益的突出问题**

　　坚持把人民群众的利益摆在首位，努力解决好事关人民群众切身利益特别是困难群众安危冷暖的突出问题。

**继续抓好生产救灾工作。**以解决好灾民吃饭问题为重点，通过生产自救、互助互济、对口支援、社会捐助、政府救济和开仓借粮等多种形式，确保春荒缺粮人口的口粮供应。加快行蓄洪区移民迁建，努力解决好灾民住房问题。积极动员和帮助灾区群众搞好农业生产，发展多种经营，多渠道增加收入，夺取生产救灾的全面胜利。

**加大扶贫开发力度。**坚持开发式扶贫方针，完善扶贫开发机制，走稳定脱贫之路。继续以山库区、沿淮行蓄洪区、江淮分水岭地区为重点，实施项目扶贫、科教扶贫和结对帮扶，加强村级基层组织建设，改善生产生活条件，提高农民自我脱贫能力。扶持80个重点贫困村实现整体脱贫。

**深入推进就业再就业工作。**坚持市场导向的就业机制，发挥政府的引导和促进作用，鼓励、支持自主创业和自谋职业。实行就业优先的产业政策和投资政策，大力发展劳动密集型企业和服务业，积极开发就业岗位。认真落实税费减免、小额信贷、岗位补贴、社会保险补贴等扶持政策，加强对下岗失业人员职业介绍、技能培训、创业咨询等服务工作，确保实现下岗失业人员再就业1.24万人以上。开展再就业援助行动，继续通过政府开发、购买公益性岗位等措施，帮助1900名大龄就业困难人员实现再就业。

**进一步完善社会保障体系。**继续做好“两个确保”工作，切实保证下岗职工基本生活费和企业离退休人员养老金按时足额发放。加强城镇“低保”规范化管理，巩固“低保”工作成果。认真落实农村“五保”工作政策，确保五保户基本生活条件。全面推进工伤保险制度，健全失业保险制度，做好事业单位和个体工商户、私营企业参保工作，推进下岗职工基本生活保障向失业保险并轨。继续完善城镇职工基本医疗保险制度改革，建立健全社会医疗救助和多层次的医疗保障体系，妥善解决困难群体医疗保障问题。进一步做好企业退休人员的社会化管理与服务工作，加强社区、街道、乡镇社会保障平台建设，健全社会保障网络。

**切实维护社会稳定。**坚持“打防结合，预防为主”的方针，进一步落实社会治安综合治理的各项措施，深入开展“强基固本年”活动，加强基层治安防控体系建设，严厉打击各种犯罪和邪教组织，制止非法宗教活动，扫除“黄、赌、毒”等社会丑恶现象。认真做好人民群众来信来访工作，健全人民内部矛盾纠纷排查调处机制，积极预防和妥善处置突发性事件。进一步落实安全生产责任制和责任追究制，防止重特大事故发生。大力整顿和规范市场秩序，组织开展食品、药品、农资、建材和烟草专卖等市场专项整治活动。

**十、全面推进精神文明建设和社会事业发展**

　　加强精神文明建设，发展各项社会事业，不断提高广大人民群众思想道德和科学文化素质，促进经济社会协调发展。

**深入开展文明创建活动。**确立抓创建促开放、抓开放促发展的观念，以“城市，让生活更美好”为主题，以争创全省文明城市创建工作先进市为目标，推动文明创建工作深入开展。认真贯彻《公民道德建设实施纲要》，以社会公德、职业道德、家庭美德教育和创优良秩序、优美环境、优质服务为主要内容，广泛开展创建文明村镇、文明社区、文明行业、文明单位和争做文明市民活动。加强公民诚信教育，在全市形成诚信为本、操守为重的良好风尚，着力打造“信用六安”。引导干部群众树立终身学习的理念，创建学习型社会。继续抓好“四五”普法教育，提高人民群众法律素质。

**实施人才强市和科教兴市战略。**牢固树立“人才是第一资源”的观念，高度重视人才工作。创新人才工作体制，建立有利于优秀人才脱颖而出、充分施展才能的选拔任用机制，不唯学历、不唯职称、不唯资历、不唯身份，不拘一格选用人才。加大人才培养和引进力度，加快造就经营管理和专业技术人才队伍。设立人才专项资金，完善人事代理制度，重奖贡献突出的人才，努力营造鼓励人才干事业、支持人才干成事业、帮助人才干好事业的社会环境，切实以事业、待遇和感情留住人才、引进人才。重视和支持引进国外智力项目工作。深化科技体制改革，开展科技特派员创业行动，支持企业博士后科研工作站建设，促进产学研联合。加大技术引进、吸收、创新、推广力度，推动科技成果向现实生产力转化。深化教育人事制度改革，巩固义务教育特别是农村义务教育成果；大力发展中等职业教育，实行基础教育、职业教育、成人教育相统筹；建立财政专项资金，资助特困家庭子女上学读书；加快实施农村中小学危房改造二期工程，改造D类危房25万平方米；鼓励社会力量办学，实现教育资源的优化配置和投入多元化。继续做好六安卫校升格为医学高等专科学校的争取工作；支持皖西学院、六安职业技术学院发展，充分发挥其对地方经济社会发展的积极作用。

**促进社会事业协调发展。**广泛开展群众性文化活动，加强农村文化、社区文化和企业文化建设，繁荣文艺创作和文化市场，依法保护和利用文物资源。推进国有文化企事业单位改革，积极发展文化产业。加强以乡镇卫生院为重点的农村卫生服务网络建设，完善疾病信息网络体系、预防控制体系和突发公共卫生医疗救助体系，提高公共卫生服务水平；开展以大病统筹为重点的新型农村合作医疗制度改革试点，提高农村医疗保健水平；继续做好非典等疫情的防控工作。广泛开展全民健身活动，办好市首届运动会。深化报业改革，加强广播电视基础设施建设，扩大信号覆盖面。发展老龄和妇女儿童事业，维护残疾人合法权益，进一步做好民族宗教、对台、气象、地震、外事、侨务、人民防空、地方志、档案等工作。组织开展经济普查。深入开展国防教育，重视加强民兵预备役工作，巩固“双拥”创建成果。

**坚持可持续发展。**统筹人与自然和谐发展，处理好经济发展同人口、资源、环境的关系。发展循环经济，制定“生态六安”建设纲要，创建国家级生态示范区，抓好退耕还林、水源涵养林建设和生态家园工程。依法保护和合理开发利用水、土地、矿产、森林资源。加强以农村、社区和流动人口为重点的计划生育管理工作，综合治理出生人口性别比失调问题，稳定低生育水平。

**十一、进一步加强政府自身建设**

　　按照“以民为本、奋发有为、清正廉洁”的要求，加快建立行为规范、运转协调、公正透明、廉洁高效的行政管理体制，建设人民满意的政府。

**强化“以民为本”的执政理念。**坚持一切想着人民，一切为了人民，一切依靠人民，始终把人民群众的呼声作为第一信号，把人民群众的满意度作为第一标准，做到权为民所用、情为民所系、利为民所谋。坚持经常深入基层，密切与人民群众的联系，建立完善市政府领导联系贫困村、企业和农村中小学校制度，把更多的时间和精力用在帮助基层和群众解决实际问题上，把更多的财力和物力用在经济社会协调发展和提高人民生活水平上。对群众反映强烈、意见集中的热点、难点问题，实行现场办公，落实解决措施。认真办好政府热线电话，坚持市长接待群众来访日制度，开设市长信箱，真心实意为群众排忧解难。

**加快政府职能转变。**按照建设服务型政府的要求，加快行政体制改革，推进政企、政事分开，切实把政府经济管理职能转到主要为市场主体服务和创造良好发展环境上来。强化政府的社会管理和公共服务职能，把属于企业的生产经营权和投资决策权还给企业，把可以由社会自我调节的职能让给社会中介组织，把群众自治范围内的事交给群众自主管理。建立和完善国有资产监督管理体制，加快实施事业单位和人事制度改革。在市直机关全面开展建设学习型、服务型、创新型机关活动。

**推进决策的民主化、科学化。**完善政府议事和决策制度。充分发挥专家决策咨询作用，凡提交政府研究决定的重大事项，先由决策咨询委员会提出建议方案。广泛开展人民建议征集活动，并做到件件有回音，事事有着落。大力推进政务公开，完善新闻发布会制度，实行重大事项社会听证制度、公示制度和办事公开制度，自觉接受人民群众监督和新闻媒体的舆论监督。

**提高依法行政水平。**全面贯彻实施《行政许可法》，清理行政许可规定和实施主体，推进相对集中行政处罚权工作。依法规范政府行为，严格按照法定权限和程序行使职权、履行职责。深入推行行政执法责任制、过错追究制和评议考核制，严格执行行政赔偿制度。加强行政执法监督和行政复议工作。自觉接受人大法律监督和政协民主监督，认真办理人大代表议案、建议和政协委员提案、建议案，主动听取各民主党派、工商联、无党派人士及各人民团体的意见和建议，积极支持工会、共青团、妇联、科协等群众团体依照法律和各自章程独立自主地开展工作。

**弘扬求真务实的工作作风。**坚持按经济规律、市场规律和科学规律办事，大兴求真务实之风，树立科学的发展观和正确的政绩观，讲实话、做实事、求实效，反对弄虚作假和形式主义，不提不切实际、哗众取宠的口号，不做急功近利、劳民伤财的事情。进一步改进政府工作流程，完善办文办事限时制，简化办事程序，提高办事效率，方便基层和群众办事；大力精简会议和文件，在去年的基础上压缩10%。加强工作目标管理，完善目标考核体系。维护政府权威，确保政令畅通。强化政府诚信建设，坚决兑现各项政策规定和服务承诺。加强政务督查，坚持以暗访和查找问题为主，确保把各项部署和措施落到实处。

**进一步加强廉政建设。**实行标本兼治、惩防并举，建立健全教育、制度、监督并重的惩治和预防腐败工作体系，着力解决损害群众利益的突出问题，全力推进政府系统反腐倡廉工作。坚持从严治政，全面落实廉政建设责任制，抓好领导干部廉洁自律，坚决查处违法违纪案件。各级政府领导干部要带头遵守和执行“四大纪律、八项要求”。进一步强化行政监察、审计等监督制约机制，建立和完善领导干部任期经济责任审计、述职述廉和重大事项报告制度。加强公务员队伍教育和管理，提高整体素质。深入开展政风行风评议活动，坚决纠正部门和行业不正之风，切实改善服务态度，提高服务质量。牢记“两个务必”，反对讲排场、比阔气、图享受等奢侈浪费现象，树立艰苦奋斗、清正廉洁的政府形象。

　　各位代表！今年是实施全面建设小康六安“三步走”战略的起始之年，我们正站在一个新的历史起点上。面对新形势、新任务，我们倍感责任重大，使命光荣而艰巨。我们坚信，在邓小平理论和“三个代表”重要思想指导下，在中共六安市委的坚强领导下，全市上下万众一心，励精图治，一定能够推动加快发展、富民强市的伟大事业，从胜利走向新的更大胜利！
